# Supplementary material for: SuPreMo: a computational tool for streamlining in silico perturbation using sequence-based predictive models
Source: Bioinformatics. 2024 May 25;40(6):btae340. doi: 10.1093/bioinformatics/btae340 (PMC11153836; doi:10.1093/bioinformatics/btae340)
Supplement: btae340_Supplementary_Data [file btae340_supplementary_data.docx]

**Supplementary Material**

Supplementary Table 1: Sequence-based predictive models and their specifications

Supplementary Table 2: SuPreMo and SuPreMo-Akita specifications.

Supplementary Figure 1: Schematic of getting sequences and maps.

Supplementary Figure 2: Schematic of the shift parameter.

Supplementary Figure 3: Example application of SuPreMo-Akita on tumor SVs.

Supplementary Text: SuPreMo input types.

### Supplementary Table 1

**Sequence-based predictive models and their specifications**

| **#** | **Model** | **Input sequence length (bp)** | **Predicted output** | **Output format** | **PMID (or DOI^b^)** |
| --- | --- | --- | --- | --- | --- |
| 1 | Akita | 1,048,576 | Contact frequency map | Matrix | 33046897 |
| 2 | Basenji | 131,072 | Various chromatin profiles (CAGE, ATACseq, ChIPseq) | Genomic tracks | 32687525 |
| 3 | Basset | 600 | DNA accessibility | Genomic tracks | 27197224 |
| 4 | Borzoi | 524 | Various chromatin profiles (CAGE, RNA-seq, DNase or ChIP-seq) | Genomic tracks | 08.30.555582 |
| 5 | BPnet | 1000 | Various chromatin profiles (TF ChIP-nexus) | Genomic tracks | 33603233 |
| 6 | C.Origami^a^ | 2,097,152 | Contact frequency map | Matrix | 36624151 |
| 7 | DanQ | 1000 | Various chromatin profiles (DNase, ChIPseq) | Genomic tracks | 27084946 |
| 8 | DeepC | 1,005,000 | Contact frequency map | Matrix | 33046896 |
| 9 | DeepFIGV | 300-2000 | Various chromatin profiles (DNase, histone ChIPseq) | Genomic tracks | 31544924 |
| 10 | DeepSea | 1000 | Various chromatin profiles (TFBS, DNase, histone ChIPseq) | Values | 26301843 |
| 11 | DeepTACT^a^ | 5,000-20,000 | Contact frequency (only for regulatory elements) | Values | 30869141 |
| 12 | Enformer | 196,608 | Various chromatin profiles (CAGE, DNase, ChIPseq) | Genomic tracks | 34608324 |
| 13 | ExPecto | 40,000 | Gene expression | Values (log RPKM) | 30013180 |
| 14 | ExPectoSC | 40,000 | Gene expression | Values (log RPKM) | 37703883 |
| 15 | HyenaDNA | 1,000-8,000 | Various chromatin profiles (TFBS, DHS, histone ChIPseq at central 200 bp) | Values | 37426456 |
| 16 | Malinois | 770,000 | Gene expression (based on CRE activity) | Values (sequence contribution scores) | 37609287 |
| 17 | ORCA | 1,000,000- 256,000,000 | Contact frequency map | Matrix | 35551308 |
| 18 | Puffin-D | 100,000 | Gene expression (transcription initiation signals) | Genomic tracks | 06.27.546584 |
| 19 | Sei | 4,000 | Various chromatin profiles (x21,907) | Sequence classes | 35817977 |
| 20 | Seq-GraphReg | 6,000,000 | Gene expression | Genomic tracks | 35396274 |
| 21 | seq2cells | 200,000 | Gene expression | Values | 07.26.550634 |
| 22 | TREDnet | 2,000 | Various chromatin profiles (TFBS, DNase, histone ChIPseq) | Values | 37603758 |
| 23 | Xpresso | 10,500 | Gene expression | Genomic tracks | 32433972 |

^a^require additional input(s)

^b^DOIs start with 10.1101/2023.

### Supplementary Table 2

**SuPreMo and SuPreMo-Akita specifications**

|  | **SuPreMo** | **SuPreMo-Akita** | **SuPreMo-Akita** |
| --- | --- | --- | --- |
| **Parameters** | --seq_len 10000  --shift -1 0 1  --revcomp add_revcomp | --augment | --augment  --get_maps  --get_tracks |
| **Number of variants** | 1000 | 100 | 100 |
| **Time (s)*** | 12 +/- 0.4 | 1,904 +/- 29.8 | 2,313 +/- 48.0 |
| **Peak memory (KB)*** | 116,805 +/- 0.4 | 526,765 +/- 7,900 | 1,772,261 +/- 4,126 |
| **Size of output (KB)** | Sequences: 83,014 | Scores: 9.5 | Maps: 638,029  Tracks: 2,935 |

*Mean +/- standard deviation across 30 runs

### Supplementary Figure 1

**Schematic of getting sequences and maps.** A: Schematic of how SuPreMo incorporates variants into a reference genome. Given a variant, SuPreMo generates reference sequence(s) (REF) and an alternate, perturbed sequence (ALT). B: Schematic of how SuPreMo-Akita adjusts contact frequency maps before comparing them to generate a perturbation score. Examples are categorized by variant type. Left panel contains maps for each allele as predicted by Akita, and the right panel shows those maps processed and ready to compare. Gray: reference genome; light blue boxes: sequences generated; white boxes: sequence deleted; dotted lines: matching positions in REF and ALT; white areas in maps: padded regions or regions with no prediction; faded areas in maps: cropped regions.

### Supplementary Figure 2

### Schematic of the shift parameter. Variants are by default centered in the generated sequences, as the deletion shown on the top. The shift parameter can be used to shift the prediction window upstream (negative shift, middle) or downstream (positive shift, bottom). A potential incentive for this is to include certain genes or regulatory elements that otherwise would not have been included in the window.

### Supplementary Figure 3


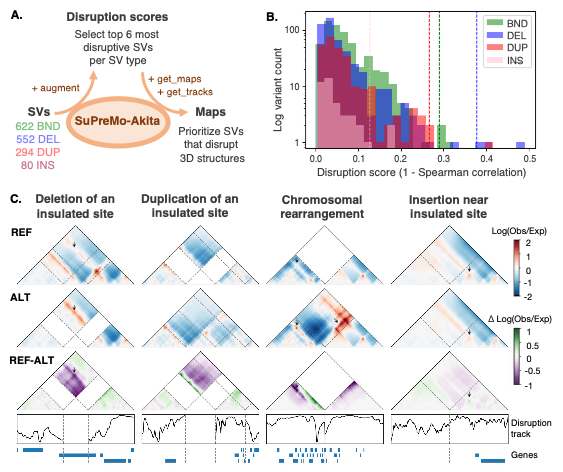


**Example application of SuPreMo-Akita on tumor SVs.** A: Schematic of scoring SVs downloaded from Talsania et al. 2022 with SuPreMo-Akita to get scores and then scoring a subset to get maps and tracks. B: Distribution of disruption scores (MSE also calculated but not shown) for SVs in A. Dashed line marks the threshold of the top 3 scoring variants for each type. C: Contact frequency maps and disruption tracks generated by SuPreMo-Akita for one selected, highly disruptive variant for each SV type. White areas in maps: padded regions or regions with no prediction; arrows: regions with changed contact.

### Supplementary Text

**SuPreMo input formats**

Suggested input types:

1. VCF file

- Following vcf 4.1/4.2 specifications, outlined here: <https://samtools.github.io/hts-specs/VCFv4.1.pdf>.

1. TXT file

- Columns required for a file with simple variants: CHROM, POS, REF, ALT.
- Columns required for a file with structural variants: CHROM, POS, REF, ALT, END, SVTYPE, SVLEN.
- SVTYPE and SVLEN refer to the type and length of structural variant.
- SVTYPE can be any of the following: DEL, DUP, INV, INS, BND.
- Column names and order must be as specified.

Additional input types:

1. BED-like file

- Columns required for simple variants: chrom, pos, end, ref, alt
- Columns required for structural variants: chrom, pos, end, ref, alt, SV type, SV length
- Exclude column names; column order must be as specified.

1. TSV file

- Following AnnotSV output format, outlined here: <https://lbgi.fr/AnnotSV/Documentation/README.AnnotSV_latest.pdf>.
